# Association Between Dietary Fiber and the Severity of Depression Symptoms

**DOI:** 10.1155/2024/5510304

**Published:** 2024-10-28

**Authors:** Yi Yang, Lubo Shi, Shihan Zeng, Chuyan Chen

**Affiliations:** Department of Gastroenterology, Beijing Friendship Hospital, Capital Medical University, State Key Laboratory for Digestive Health, National Clinical Research Center for Digestive Diseases, Beijing 100050, China

**Keywords:** correlation, depression symptoms, dietary fiber, National Health and Nutrition Examination Survey (NHANES)

## Abstract

**Aim:** Our study is aimed at exploring the correlation between consumption of dietary fiber and the severity of depression symptoms.

**Methods:** This study utilized data from the National Health and Nutrition Examination Survey spanning from 2007 to 2018, employing a cross-sectional design. The relationship between the severity of depression symptoms and intake of total cereals, vegetables, and fruits dietary fiber was assessed using both univariate and multivariate linear/logistic regression analyses. Stratified analyses were conducted based on hypertension, diabetes, dyslipidemia, cancer or malignancy, and cardiovascular disease.

**Results:** This study included 28,852 participants who were classified into 21,696 with nondepression symptoms, 4614 with mild depression symptoms, 1583 with moderate depression symptoms, 684 with moderately severe depression symptoms, and 275 with severe depression symptoms. After adjusting all confounding factors, we observed a negative correlation between total dietary fiber and depression symptoms (beta = −0.004, 95% confidence intervals [CIs]: −0.006, −0.002). Taking nondepression symptoms as a reference, total dietary fiber was found to have an inverse association with moderate (OR = 0.976, 95% CI: 0.962–0.991), moderately severe (OR = 0.963, 95% CI: 0.938–0.990), and severe depression symptoms (OR = 0.960, 95% CI: 0.921–1.001; marginal significance), respectively.

**Conclusion:** The intakes of total dietary fibers might be related to moderate/moderately severe/severe depression symptoms, and a negative association was shown between total dietary fiber intakes and the risk of depression symptoms.

## 1. Introduction

Depression symptoms are a prevalent mental disorder that significantly contributes to the global burden of disease [1]. In 2020, an estimated 246 million individuals worldwide suffered from depression symptoms, and its prevalence rate is projected to continue increasing [2]. In consequence, it is crucial to give careful consideration about the risk and prevention of depression symptoms.

Altered neurotransmission, inflammation, and reduced neuroplasticity were considered as possible pathophysiological mechanisms related to depression symptoms [3, 4]. At present, pharmacotherapy may be an effective treatment for depression symptoms, but the efficacy of medication appears to be limited to patients with major depression symptoms, and its impact on suicide risk remains unknown [5]. An increasing number of reports have emphasized that nutrients enhance the synthesis of neurotransmitters involved in regulating mood, appetite, and cognition, thereby alleviating the development of depression symptoms [6–8]. Dietary fiber has been widely recognized as an important component of a healthy diet and a major regulator of intestinal microbial composition [9]. Previous studies have indicated that the brain-gut axis could be influenced by dietary fiber, potentially contributing to the regulation of mental disorders [10, 11]. Nevertheless, the relationship of dietary fiber with depression remains a topic of controversy in current research. A study performed among the general adult population in Tianjin, China, showed that a higher intake of vegetable and soya fiber had a decreased likelihood of depressive symptoms [12]. Xu et al. proposed that there exists an inverse relationship between the consumption of fiber from overall, vegetable, and fruit sources and the manifestation of depressive symptoms in adult individuals [13]. However, a study conducted on Japanese workers using cross-sectional analysis did not discover a correlation between overall fiber consumption and the likelihood of experiencing depressive symptoms [14]. In general, the association between depression and dietary fiber consumption may vary depending on the population and type of fiber source used.

Herein, the aim of this study was to assess the correlation between the consumption of dietary fiber from commonly ingested foods and the extent of depressive symptoms in a considerably large sample obtained from the 2007 to 2018 National Health and Nutrition Examination Survey (NHANES).

## 2. Methods

### 2.1. Data Sources

In this cross-sectional study, all data used and analyzed were sourced from NHANES, a program established by the National Center for Health Statistics (NCHS) with the objective of evaluating the health condition and nutritional status of the American population [15]. The survey employs a complex and multistage probabilistic sampling approach to acquire a nationally representative sample [15]. The NCHS Research Ethics Review Board reviewed and approved the NHANES study, and all participants gave written informed consent.

Inclusion criteria are as follows: individuals who possess comprehensive data on dietary fiber and symptoms of depression. Exclusion criteria are as follows: (1) participants < 18 years old, (2) pregnant participants and lactating women, (3) female participants whose total energy intake per day is below 500 or exceeds 5000 kcal, (4) male participants whose maximum total energy intake per day is below 500 or exceeds 8000 kcal, and (5) participants with a lack of baseline information. All methods of this study adhered to the guidelines in the Helsinki Declaration.

### 2.2. Data Collection

A number of baseline characteristics of all participants were collected: age, sex, marital status, race, education level, body mass index (BMI, kg/m^2^), drinking, smoking, poverty-to-income ratio (PIR), physical activity (metabolic equivalent [MET]⸳minute per week), dyslipidemia, diabetes, hypertension, cancer or malignancy, liver disease, cardiovascular disease (CVD), seen a mental health professional in the prior 12 months, antidepression drug, energy intakes (kilocalories), protein (grams), total fat (grams), carbohydrate (grams), iron, calcium (milligrams), potassium (milligrams), sodium (milligrams), total saturated fatty acids (grams), total polyunsaturated fatty acids (PUFAs, grams), total monounsaturated fatty acids (MUFAs, g), vitamin B_12_ (milligrams), vitamin B_6_ (milligrams), caffeine (milligrams), dietary fiber (grams), cereal fiber (grams), vegetable fiber (grams), and fruit fiber (grams). The MET is commonly utilized to quantify the energy expenditure during a specific activity, and physical activity is determined: MET × exercise duration per week (minutes per week) of the corresponding activity.

### 2.3. Definition of Depression Symptoms

In the NHANES database, the depression symptoms of the participants were evaluated using a screening tool called the 9-item Patient Health Questionnaire (PHQ-9), which provides a total score ranging from 0 to 27 [16]. The scores were calculated based on the participants' self-reported occurrence of depressive symptoms within the previous fortnight [17]. Assessments of depression symptoms severity were assigned based on PHQ-9 scores: *none/minimal* = scores ranging from 0 to 4, *mild symptoms* = scores ranging from 5 to 9, *moderate symptoms* = scores ranging from 10 to 14, *moderately severe symptoms* = scores ranging from 15 to 19, and *severe symptoms* = scores ranging from 20 to 27 [18].

### 2.4. Dietary Fiber Intakes

Participants' information on diet in the NHANES database was collected through two 24-h dietary recall interviews [19]. The first diet interview was conducted at the mobile examination center (MEC), followed by a second interview obtained via telephone 3–10 days later. Dietary fiber from cereal, fruits, and vegetables was calculated according to the US Department of Agriculture's Dietary Research Food and Nutrition Database for Dietary Studies [20]. Dietary fiber obtained from two 24-h dietary recall interviews was averaged. Total dietary fiber was classified into three groups based on the tertiles: Q1: <11.95 g; Q2: 11.95–18.70 g; Q3: ≥ 18.70 g. Vegetable fiber was initially categorized based on the zero intake and nonzero intake. Vegetables fiber was initially categorized based on the zero intake (0 g) and nonzero intake. Subsequently, within the nonzero group, vegetable fiber levels were further divided into two groups based on the median value (0–2.20 ≥ 2.20 g). Similarly, cereal fiber levels were divided into three groups: 0, 0–2.30, and ≥ 2.30 g. Fruit fiber levels were divided into three groups: 0, 0–0.70, and ≥ 0.70 g.

### 2.5. Statistical Analysis

The continuous variables were performed by weightedmean ± standard error(SE) using the number of cases and composition ratio (*n*[%]) to explore the distribution of the categorical variables. Differences in the distribution of variables between groups adopted weighted chi-square and weighted *t*-test. Considering the complex sampling design of NHANES, weights were taken into account for all descriptive indicators and tests.

The analysis employed a weighted univariate linear regression approach to identify potential confounding factors (Table S1). A significance level of *p* < 0.05 was used to determine statistical significance. We employed weight univariate and multivariate linear regression analyses to evaluate the correlation between depression symptoms and the consumption of total fiber, cereal fiber, vegetable fiber, and fruit fiber. We adopted weight univariate and multivariate logistic regression to evaluate the association between the severity of depression symptoms and the intakes of total fiber, cereal fiber, vegetable fiber, and fruit fiber. Model 1 was a crude model. Model 2 adjusted for sex, race, education level, marital status, BMI, smoking, PIR, physical activity, hypertension, diabetes, dyslipidemia, liver disease, CVD, seen a mental health professional in the prior 12 months, antidepression drug, energy, carbohydrate, protein, total fat, iron, calcium, potassium, sodium, total saturated fatty acids, MUFA, PUFA, vitamin B_6_, and caffeine. Stratified analyses were also performed based on hypertension, diabetes, dyslipidemia, cancer or malignancy, and CVD to evaluate the correlation between the severity of depression symptoms and the intakes of total fiber, cereal fiber, vegetable fiber, and fruit fiber. Weighted beta, odds ratio (OR), and 95% confidence interval (CI) were computed. SAS 9.4 software performed statistical analysis, and R 4.2.0 software was used to draw the stacked bar chart.

## 3. Results

### 3.1. Characteristics

**Figure 1:**
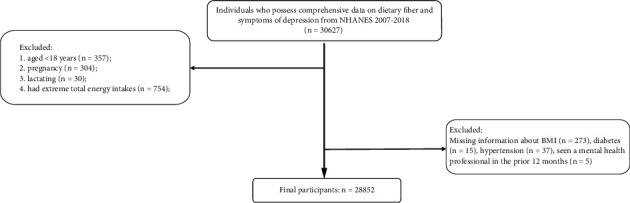
Flowchart for participant selection from NHANES.

After removing certain individuals who were aged < 18 years (*n* = 357), were pregnant (*n* = 304), were lactating (*n* = 30), had extreme total energy intakes (*n* = 754), and had the missing information on baseline information (*n* = 330), this study ultimately contained 28,852 eligible participants (Figure 1).  Table 1 summarizes the baseline information of all eligible participants. The individuals were divided into five categories: nondepression symptoms (*n* = 21,696), mild depression symptoms (*n* = 4614), moderate depression symptoms (*n* = 1583), moderately severe depression symptoms (*n* = 684), and severe depression symptoms (*n* = 275). Compared with nondepression symptoms participants, participants with depression symptoms (mild/moderate/moderately severe/severe) appeared to have lower intakes of energy, protein, total fat, and iron.

### 3.2. The Relationship Between the Depression Symptoms and Total Fiber Intakes of Cereals, Vegetables, and Fruits

According to the findings presented in Table 2, after adjusting for all potential confounding variables, negative correlations were observed between the consumption of total fiber and symptoms of depression (beta = −0.004, 95% CI: −0.006, −0.002; *p* < 0.001), as well as between the consumption of vegetable fiber and symptoms of depression (beta = −0.007, 95% CI: −0.013, −0.001; *p* = 0.015). However, the relationship of the intakes of cereals and fruit fiber with the risk of depression symptoms did not show any significant difference after adjusting for confounding factors (Model 2). When considering dietary fiber as a categorical variable, we only found that higher total fiber was associated with decreased risk of depression symptoms (total fiber 11.95–18.70 g: beta = −0.059, 95% CI: −0.089, −0.028, *p* < 0.001; total fiber ≥ 18.70 g: beta = −0.088, 95% CI: −0.126, −0.051; *p* < 0.001).

Similarly, weighted OR and 95% CI in Table 3 showed an association between the severity of depression symptoms and intakes of total cereal, vegetable, and fruit fiber. In this crude model, taking nondepression symptoms as reference, total dietary fiber was found to be associated with mild (OR = 0.983, 95% CI: 0.978–0.988), moderate (OR = 0.958, 95% CI: 0.949–0.968), moderately severe (OR = 0.947, 95% CI: 0.931–0.963), and severe depression symptoms (OR = 0.941, 95% CI: 0.918–0.964), respectively. After adjusting for all confounding factors, total dietary fiber still remained a protective factor for moderate (OR = 0.976, 95% CI: 0.962–0.991), moderately severe (OR = 0.963, 95% CI: 0.938–0.990), and severe depression symptoms (OR = 0.960, 95% CI: 0.921–1.001; marginal significance) depression symptoms. Additionally, we obtained that the OR was 0.909 (95% CI: 0.840–0.983) in the association of vegetable fiber and moderate depression symptoms, which implied that vegetable fiber intakes might be associated with a protective effect of patients with moderate depression symptoms. When considering dietary fiber as a categorical variable, we also found the associations between total fiber and the severity of depression symptoms (Table 3).

### 3.3. Stratified Analyses

We performed the subgroup analyses based on populations with different histories of diseases (Table 4). For patients without cancer, CVD, dyslipidemia, or hypertension, total dietary fiber was found to be linked to a reduced likelihood of experiencing moderate and moderately severe depression symptoms, while the relationship between total dietary fiber and moderate depression symptoms was also observed in patients with diabetes. Similarly, among patients without cancer, or CVD, total dietary fiber was found to have a potential protective effect against severe depression symptoms. Additionally, the relationship between the intakes of cereals, vegetables, and fruit fiber and the severity of depression symptoms among different subgroups was obtained in Table 3.

## 4. Discussion

Large samples were used in the current study to seek the potential link between dietary fibers from cereals, vegetables, and fruits and the severity of depression symptoms. The findings expounded a negative correlation between total dietary fiber intake and depression symptoms. In addition, there was an observed correlation between the consumption of overall dietary fiber and symptoms of depression ranging from moderate to moderately severe.

Previous studies mostly focused their attention on the correlation between dietary fiber intake and depression symptoms [11, 21, 22]. Most studies have acknowledged that dietary fiber (an essential part of a healthy diet) intake appears to be related to a decreased risk of depression symptoms [12, 23, 24]. Nowadays, the mechanism between dietary fiber and depression symptoms remains poorly understood, but some possible explanations have been proposed: the brain-gut axis enables the interaction between the intestinal microbiota and the brain, which plays a role in regulating various aspects of brain function such as mood, behavior, and cognitive abilities, thereby decreasing the risk of depression symptoms. Dietary fiber has the potential to efficiently control the composition of intestinal microbiota [9, 25]. Short-chain fatty acids, as neuroactive bacterial metabolites, were produced by dietary fibers and have been pointed out that could regulate inflammatory responses, which might modulate brain function and improve depression symptoms [14, 26].

Patients with different levels of depression symptoms might have different eating habits. However, the effect of dietary fiber on American patients with varying severity of depression symptoms is still poorly understood to date. In this present study, we considered dividing the depressed American patients into four groups according to the severity (mild/moderate/moderately severe/severe). The result showed the total fiber intakes were related to moderate/moderately severe depression symptoms. It is widely acknowledged that cereals, vegetables, and fruits are rich sources of dietary fiber. Extensive literature has reported the antioxidant effects of a diet pattern abundant in these food groups on mental health. However, in contrast to prior research findings, we found no statistically significant difference in the association between the intakes of fiber from cereals and fruits and depression symptoms in the present study. Notably, some relationships between fiber from cereals, vegetables, and fruits and depression symptoms in the subgroup analyses based on populations with different histories of diseases were observed. This could potentially be attributed to the dietary patterns of the American population. Additional research is required to explore the role of dietary fiber intake for patients with depression symptoms.

Overall, the collective findings suggest that dietary fiber intakes may mitigate the likelihood of developing depressive symptoms. However, there are also some inevitable limitations. First of all, it should be information bias. Participants' information in this study was all downloaded from the NHANES database, and dietary fiber intakes were recorded relying on two 24-h dietary recall interviews. Secondly, because of the design of this cross-sectional study, causal interpretations cannot be obtained for the correlation between the severity of depression symptoms and dietary fiber intakes. Although we speculated that total dietary fiber intakes may be associated with depression symptoms, we must acknowledge the possibility of reverse causation. It is possible that depressed patients may change their eating habits, such as eating more ultraprocessed foods, which reduces the intake of dietary fiber. More prospective studies are required to confirm the relationship. Thirdly, the severity of depression symptoms for participants was assessed by using the PHQ-9 in this study. Although PHQ-9 is regarded as a valid tool in evaluating the severity of depression symptoms, it is inevitable that some patients may get different results from a psychiatrist's diagnosis [27, 28]. Dietary fiber generally can be categorized into soluble and insoluble fiber [26]. Due to all data utilized in this study originating from the NHANES database, we were unable to estimate the relationship between depression symptoms and the two fibers, which may bring out different impacts on depression symptom risk. Lastly, despite adjusting for potential confounders, our study may be influenced by unmeasured variables in the NHANES database, such as cognitive–behavioral therapy, interpersonal therapy, and meditation. Further and prospective studies should be completed in the future.

## 5. Conclusion

In summary, intake of total dietary fiber might be associated with moderate/moderately severe/severe depression symptoms. In addition, the result showed an inverse correlation between total dietary fiber and the likelihood of depression symptoms. However, these findings should be confirmed by more prospective studies in the future.

## Figures and Tables

**Table 1:** Baseline characteristics of participants according to the severity of depression symptoms.

**Variables**	**Total (*n* = 28687)**	**Nondepression symptom group (*n* = 12,004)**	**Mild depression symptom group (*n* = 13,509)**	**Moderate depression symptom group (*n* = 1943)**	**Moderately severe depression symptom group (*n* = 849)**	**Severe depression symptom group (*n* = 382)**	**p**
Age (years), mean (SE)	46.81 (0.25)	46.95 (0.28)	46.45 (0.42)	45.75 (0.53)	46.32 (0.80)	47.72 (1.20)	0.091
Gender, male, *n* (%)	14,561 (50.01)	11,624 (52.76)	1991 (42.73)	612 (37.49)	236 (35.55)	98 (38.99)	< 0.001
Race, *n* (%)							0.003
White	12,080 (67.65)	9055 (68.27)	1955 (66.65)	663 (63.80)	277 (62.91)	130 (63.79)	
Black	6128 (10.80)	4593 (10.39)	1000 (11.80)	338 (12.79)	146 (13.54)	51 (11.90)	
Other	10,644 (21.54)	8048 (21.34)	1659 (21.55)	582 (23.41)	261 (23.55)	94 (24.31)	
Education level, *n* (%)							< 0.001
Less than high school	6336 (14.32)	4343 (12.80)	1142 (17.05)	494 (21.74)	254 (27.63)	103 (28.67)	
Above high school	21,030 (82.36)	16,247 (83.91)	3198 (79.30)	1014 (74.66)	405 (69.89)	166 (70.23)	
Unknown	1486 (3.33)	1106 (3.29)	274 (3.65)	75 (3.60)	25 (2.48)	6 (1.10)	
Marital status, *n* (%)							< 0.001
Married	14,044 (53.50)	11,296 (56.87)	1889 (45.22)	550 (37.22)	225 (36.13)	84 (32.15)	
Unmarried	13,329 (43.19)	9296 (39.86)	2454 (51.11)	960 (59.28)	434 (61.40)	185 (66.75)	
Unknown	1479 (3.31)	1104 (3.27)	271 (3.67)	73 (3.50)	25 (2.48)	6 (1.10)	
PIR, *n* (%)							< 0.001
< 1	5661 (13.26)	3610 (10.82)	1172 (18.56)	521 (24.98)	258 (30.77)	100 (29.58)	
≥ 1	20,689 (79.78)	16,186 (82.18)	3062 (74.40)	937 (68.82)	357 (61.63)	147 (64.60)	
Unknown	2502 (6.97)	1900 (7.00)	380 (7.03)	125 (6.20)	69 (7.60)	28 (5.82)	
Smoking, yes, *n* (%)	12342 (44.04)	8688 (41.01)	2202 (50.98)	874 (59.10)	394 (60.30)	184 (69.13)	< 0.001
Drinking, *n* (%)							0.292
No	7227 (19.72)	5529 (20.03)	1085 (18.27)	384 (19.91)	160 (17.72)	69 (20.91)	
No	21,107 (79.14)	15,791 (78.86)	3426 (80.33)	1170 (79.13)	516 (81.31)	204 (77.93)	
Unknown	518 (1.14)	376 (1.11)	103 (1.40)	29 (0.96)	8 (0.97)	2 (1.17)	
BMI (kg/m^2^), mean (SE)	29.06 (0.09)	28.70 (0.09)	30.06 (0.17)	30.49 (0.27)	31.56 (0.47)	30.19 (0.45)	< 0.001
Physical activity (MET⁣^∗^min/week), mean (SE)	922.94 (12.58)	908.11 (15.62)	966.14 (17.12)	831.13 (34.12)	722.43 (54.51)	692.26 (78.14)	< 0.001
< 750	12,314 (38.27)	8845 (36.26)	2099 (42.03)	798 (49.03)	397 (52.63)	175 (57.15)	
≥ 750	16,532 (61.72)	12,848 (63.73)	2512 (57.93)	785 (50.97)	287 (47.37)	100 (42.85)	
Unknown	6 (0.01)	3 (0.01)	3 (0.03)	0 (0.00)	0 (0.00)	0 (0.00)	
Diabetes, yes, *n* (%)	3649 (9.53)	2439 (8.42)	716 (12.09)	280 (14.67)	150 (16.13)	64 (18.12)	< 0.001
Hypertension, yes, *n* (%)	10,137 (31.53)	7115 (29.35)	1845 (36.74)	685 (39.96)	343 (46.72)	149 (50.82)	< 0.001
Dyslipidemia, *n* (%)							< 0.001
No	15,692 (57.05)	12,169 (58.34)	2354 (53.86)	731 (50.89)	316 (51.34)	122 (46.20)	
Yes	9439 (32.43)	6792 (31.31)	1637 (35.33)	603 (36.57)	285 (38.13)	122 (44.45)	
Unknown	3721 (10.52)	2735 (10.35)	623 (10.80)	249 (12.54)	83 (10.53)	31 (9.35)	
Cancer or malignancy, *n* (%)							0.397
No	24,641 (86.36)	18,570 (86.44)	3911 (86.09)	1351 (86.33)	581 (86.42)	228 (84.08)	
Yes	2726 (10.28)	2022 (10.25)	428 (10.16)	159 (10.16)	78 (11.10)	39 (14.46)	
Unknown	1485 (3.36)	1104 (3.31)	275 (3.76)	73 (3.50)	25 (2.48)	8 (1.46)	
Liver condition, *n* (%)							< 0.001
No	26,205 (93.06)	19,906 (93.78)	4097 (91.74)	1386 (88.84)	584 (88.45)	232 (87.00)	
Yes	1135 (3.52)	674 (2.88)	235 (4.44)	120 (7.48)	72 (8.59)	34 (10.79)	
Unknown	1512 (3.42)	1116 (3.34)	282 (3.82)	77 (3.68)	28 (2.96)	9 (2.20)	
CVD, yes, *n* (%)	2915 (8.16)	1846 (6.88)	602 (10.97)	241 (12.83)	142 (16.59)	84 (27.10)	< 0.001
Seen mental health professional in the prior 12 months, yes, *n* (%)	2373 (8.75)	1076 (5.56)	608 (14.41)	358 (24.49)	213 (34.43)	118 (44.61)	< 0.001
Antidepression drug, yes, *n* (%)	3038 (13.02)	1428 (8.93)	805 (21.74)	462 (35.05)	229 (36.17)	114 (44.89)	< 0.001
Energy (kcal), mean (SE)	2123.57 (7.19)	2135.15 (8.00)	2111.74 (16.97)	2053.86 (25.66)	1980.44 (40.38)	2026.36 (80.89)	< 0.001
Protein (g), mean (SE)	83.03 (0.34)	84.27 (0.36)	80.94 (0.77)	76.08 (1.19)	73.08 (1.89)	72.09 (4.00)	< 0.001
Carbohydrate (g), mean (SE)	252.20 (0.91)	253.12 (1.02)	249.52 (1.90)	249.85 (3.58)	242.57 (4.46)	253.85 (7.32)	0.103
Total fat (g), mean (SE)	82.43 (0.38)	82.84 (0.41)	82.74 (0.93)	79.52 (1.27)	75.15 (2.02)	73.40 (2.72)	< 0.001
Iron, mean (SE)	14.98 (0.07)	15.18 (0.08)	14.63 (0.14)	13.86 (0.27)	13.29 (0.37)	13.50 (0.66)	< 0.001
Calcium (mg), mean (SE)	972.45 (5.60)	980.86 (6.18)	962.29 (11.56)	931.43 (15.84)	861.37 (21.02)	891.40 (56.66)	< 0.001
Potassium (mg), mean (SE)	2683.05 (13.71)	2727.53 (14.17)	2589.93 (21.91)	2441.66 (34.85)	2406.33 (61.06)	2376.94 (102.81)	< 0.001
Sodium (mg), mean (SE)	3523.14 (13.43)	3554.08 (14.43)	3507.57 (36.25)	3285.31 (46.93)	3184.99 (93.21)	3162.41 (118.84)	< 0.001
Total saturated fatty acids (gm), mean (SE)	26.91 (0.15)	26.92 (0.16)	27.33 (0.35)	26.66 (0.44)	24.85 (0.71)	24.62 (1.03)	0.015
MUFA (gm), mean (SE)	29.22 (0.14)	29.42 (0.15)	29.22 (0.33)	27.91 (0.49)	26.38 (0.75)	25.46 (0.97)	< 0.001
PUFA (gm), mean (SE)	18.82 (0.09)	18.99 (0.11)	18.71 (0.23)	17.68 (0.34)	17.04 (0.59)	16.71 (0.67)	< 0.001
Vitamin B_6_ (mg), mean (SE)	2.16 (0.01)	2.18 (0.01)	2.12 (0.04)	2.05 (0.08)	1.85 (0.06)	2.13 (0.16)	< 0.001
Vitamin B_12_ (mg), mean (SE)	6.32 (0.06)	6.31 (0.07)	6.38 (0.16)	6.47 (0.35)	5.83 (0.41)	6.15 (0.66)	0.750
Caffeine (mg), mean (SE)	168.57 (2.64)	165.69 (2.74)	171.11 (4.73)	190.43 (8.62)	192.07 (13.26)	203.40 (19.92)	0.014
Dietary fiber (g), mean (SE)	17.10 (0.13)	17.54 (0.13)	16.22 (0.19)	14.72 (0.27)	14.14 (0.44)	13.83 (0.57)	< 0.001
Dietary fiber (g), *n* (%)							< 0.001
< 11.95	9590 (30.86)	6758 (28.81)	1711 (34.94)	665 (41.44)	312 (45.10)	144 (48.07)	
11.95–18.70	9616 (34.44)	7278 (34.60)	1541 (34.26)	505 (33.85)	219 (33.09)	73 (29.53)	
≥ 18.70	9646 (34.70)	7660 (36.59)	1362 (30.80)	413 (24.72)	153 (21.80)	58 (22.39)	
Cereal fiber (g), mean (SE)	0.33 (0.02)	0.34 (0.02)	0.32 (0.03)	0.28 (0.07)	0.12 (0.03)	0.14 (0.09)	< 0.001
Cereal fiber (g), *n* (%)							0.038
0	26,517 (91.53)	19,912 (91.36)	4250 (91.51)	1447 (92.36)	646 (94.20)	262 (95.96)	
0–2.30	1156 (4.04)	866 (4.08)	182 (3.98)	74 (3.76)	25 (4.33)	9 (2.12)	
≥ 2.30	1179 (4.44)	918 (4.56)	182 (4.51)	62 (3.87)	13 (1.47)	4 (1.92)	
Vegetable fiber (g), mean (SE)	0.28 (0.01)	0.29 (0.02)	0.27 (0.02)	0.16 (0.02)	0.15 (0.04)	0.17 (0.05)	< 0.001
Vegetable fiber (g), *n* (%)							0.022
0	26,097 (90.58)	19,558 (90.25)	4199 (90.87)	1456 (93.08)	636 (94.37)	248 (91.23)	
0–2.20	1371 (4.95)	1047 (5.07)	211 (4.87)	66 (3.92)	28 (3.31)	19 (5.89)	
≥ 2.20	1384 (4.47)	1091 (4.68)	204 (4.26)	61 (3.00)	20 (2.32)	8 (2.88)	
Fruit fiber (g), mean (SE)	0.21 (0.01)	0.21 (0.01)	0.20 (0.02)	0.15 (0.03)	0.18 (0.04)	0.06 (0.02)	< 0.001
Fruit fiber (g), *n* (%)							0.008
0	24,672 (85.86)	18,458 (85.50)	3979 (86.39)	1383 (87.84)	598 (87.34)	254 (94.06)	
0–0.70	2052 (6.49)	1580 (6.63)	302 (5.89)	115 (6.81)	44 (6.12)	11 (3.03)	
≥ 0.70	2128 (7.66)	1658 (7.87)	333 (7.72)	85 (5.35)	42 (6.54)	10 (2.91)	

*Note:* The asterisk (⁣^∗^) means multiplication. MET⁣^∗^min/week represents MET multiplied by min and divided by week.

Abbreviations: BMI = body mass index, CVD = cardiovascular disease, MET = metabolic equivalent, MUFA = total monounsaturated fatty acids, PIR = poverty-to-income ratio, PUFA = total polyunsaturated fatty acids.

**Table 2:** Weight univariate and multivariate linear regression analyses.

**Variables**	**Model 1**		**Model 2**	
	**Beta (95% CI)**	**p**	**Beta (95% CI)**	**p**
Total fiber	−0.008 (−0.009, −0.007)	< 0.001	−0.004 (−0.006, −0.002)	< 0.001
Total fiber				
< 11.95 g	Ref		Ref	
11.95–18.70 g	−0.109 (−0.139, −0.080)	< 0.001	−0.059 (−0.089, −0.028)	< 0.001
≥ 18.70 g	−0.179 (−0.205, −0.153)	< 0.001	−0.088 (−0.126, −0.051)	< 0.001
Cereal fiber	−0.008 (−0.012, −0.003)	0.003	−0.002 (−0.006, 0.003)	0.458
Cereal fiber				
0 g	Ref		Ref	
0–2.30 g	−0.024 (−0.069, 0.021)	0.305	−0.008 (−0.050, 0.034)	0.711
≥ 2.30 g	−0.073 (−0.106, −0.039)	< 0.001	−0.026 (−0.061, 0.009)	0.147
Vegetable fiber	−0.014 (−0.020, −0.009)	< 0.001	−0.007 (−0.013, −0.001)	0.015
Vegetable fiber				
0 g	Ref		Ref	
0–2.20 g	−0.043 (−0.085, −0.001)	0.050	−0.028 (−0.073, 0.016)	0.219
≥ 2.20 g	−0.086 (−0.128, −0.044)	< 0.001	−0.035 (−0.079, 0.008)	0.114
Fruit fiber	−0.015 (−0.025, −0.005)	0.004	−0.003 (−0.013, 0.007)	0.511
Fruit fiber				
0 g	Ref		Ref	
0–0.70 g	−0.037 (−0.080, 0.006)	0.097	−0.028 (−0.073, 0.016)	0.214
≥ 0.70 g	−0.064 (−0.101, −0.028)	< 0.001	−0.033 (−0.075, 0.009)	0.124

*Note:* Model 1: unadjusted. Model 2: adjusted gender, race, education level, marital status, body mass index, smoking, poverty-to-income ratio, physical activity, hypertension, diabetes, dyslipidemia, liver disease, cardiovascular disease, seen a mental health professional in the prior 12 months, antidepression drug, energy, protein, total fat, iron, calcium, potassium, sodium, saturated fatty acids, total monounsaturated fatty acids, total polyunsaturated fatty acids, vitamin B_6_, and caffeine.

Abbreviation: CI = confidence interval.

**Table 3:** Relationship between the depression symptoms and total fiber intakes of cereals, vegetables, and fruits.

**Variables**	**Depression symptoms**	**Model 1**		**Model 2**	
		**OR (95% CI)**	**p**	**OR (95% CI)**	**p**
Total fiber	None/mild	0.983 (0.978–0.988)	< 0.001	0.993 (0.984–1.002)	0.146
	None/moderate	0.958 (0.949–0.968)	< 0.001	0.976 (0.962–0.991)	0.003
	None/moderately severe	0.947 (0.931–0.963)	< 0.001	0.963 (0.938–0.990)	0.007
	None/severe	0.941 (0.918–0.964)	< 0.001	0.960 (0.921–1.001)	0.058

Total fiber					

< 11.95 g		Ref		Ref	

11.95–18.70 g	None/mild	0.816 (0.737–0.904)	< 0.001	0.881 (0.788–0.984)	0.026
	None/moderate	0.680 (0.581–0.797)	< 0.001	0.827 (0.675–1.014)	0.068
	None/moderately severe	0.611 (0.470–0.794)	< 0.001	0.760 (0.535–1.078)	0.122
	None/severe	0.512 (0.376–0.696)	< 0.001	0.621 (0.399–0.964)	0.034

≥ 18.70 g	None/mild	0.694 (0.627–0.768)	< 0.001	0.826 (0.712–0.957)	0.012
	None/moderate	0.470 (0.400–0.552)	< 0.001	0.690 (0.535–0.892)	0.005
	None/moderately severe	0.381 (0.289–0.502)	< 0.001	0.546 (0.344–0.866)	0.011
	None/severe	0.367 (0.250–0.539)	< 0.001	0.522 (0.280–0.972)	0.041

Cereal fiber	None/mild	0.993 (0.971–1.016)	0.550	1.012 (0.991–1.033)	0.275
	None/moderate	0.981 (0.928–1.036)	0.486	1.007 (0.954–1.063)	0.794
	None/moderately severe	0.833 (0.741–0.937)	0.003	0.846 (0.743–0.964)	0.013
	None/severe	0.874 (0.666–1.147)	0.328	0.908 (0.698–1.181)	0.467

Cereal fiber					

0 g		Ref		Ref	

0–2.30 g	None/mild	0.974 (0.786–1.208)	0.811	1.027 (0.826–1.278)	0.806
	None/moderate	0.913 (0.699–1.192)	0.500	0.964 (0.736–1.264)	0.790
	None/moderately severe	1.030 (0.591–1.795)	0.916	1.108 (0.548–2.242)	0.773
	None/severe	0.496 (0.280–0.879)	0.017	0.526 (0.249–1.108)	0.090

≥ 2.30 g	None/mild	0.987 (0.801–1.216)	0.898	1.163 (0.902–1.500)	0.241
	None/moderate	0.840 (0.628–1.122)	0.235	0.986 (0.739–1.315)	0.922
	None/moderately severe	0.313 (0.166–0.590)	< 0.001	0.343 (0.171–0.688)	0.003
	None/severe	0.400 (0.123–1.299)	0.126	0.459 (0.143–1.470)	0.187

Vegetable fiber	None/mild	0.987 (0.955–1.020)	0.421	1.003 (0.968–1.039)	0.867
	None/moderate	0.884 (0.826–0.946)	< 0.001	0.909 (0.840–0.983)	0.018
	None/moderately severe	0.871 (0.761–0.998)	0.046	0.908 (0.785–1.050)	0.189
	None/severe	0.899 (0.774–1.045)	0.163	0.956 (0.835–1.094)	0.506

Vegetable fiber					

0 g		Ref		Ref	

0–2.20 g	None/mild	0.955 (0.760–1.201)	0.691	0.982 (0.764–1.262)	0.886
	None/moderate	0.750 (0.516–1.090)	0.130	0.747 (0.489–1.140)	0.174
	None/moderately severe	0.625 (0.404–0.966)	0.035	0.608 (0.380–0.971)	0.037
	None/severe	1.151 (0.727–1.822)	0.546	1.271 (0.777–2.081)	0.336

≥ 2.20 g	None/mild	0.904 (0.724–1.128)	0.368	1.000 (0.786–1.272)	0.997
	None/moderate	0.622 (0.423–0.914)	0.016	0.749 (0.473–1.189)	0.217
	None/moderately severe	0.475 (0.254–0.889)	0.020	0.623 (0.319–1.216)	0.163
	None/severe	0.609 (0.240–1.546)	0.293	0.936 (0.356–2.462)	0.892

Fruit fiber	None/mild	0.979 (0.936–1.024)	0.355	1.010 (0.966–1.057)	0.660
	None/moderate	0.895 (0.780–1.028)	0.114	0.929 (0.789–1.093)	0.369
	None/moderately severe	0.954 (0.840–1.084)	0.468	0.988 (0.849–1.150)	0.878
	None/severe	0.547 (0.328–0.912)	0.021	0.548 (0.317–0.949)	0.032

Fruit fiber					

0 g		Ref		Ref	

0–0.70 g	None/mild	0.879 (0.727–1.064)	0.184	0.887 (0.713–1.104)	0.280
	None/moderate	1.000 (0.775–1.290)	0.999	1.020 (0.765–1.358)	0.893
	None/moderately severe	0.905 (0.609–1.343)	0.615	0.880 (0.548–1.413)	0.595
	None/severe	0.416 (0.223–0.778)	0.006	0.434 (0.215–0.879)	0.021

≥ 0.70 g	None/mild	0.971 (0.829–1.136)	0.706	1.044 (0.876–1.244)	0.628
	None/moderate	0.662 (0.469–0.934)	0.019	0.719 (0.478–1.081)	0.112
	None/moderately severe	0.813 (0.523–1.263)	0.354	0.871 (0.516–1.471)	0.602
	None/severe	0.336 (0.155–0.727)	0.006	0.317 (0.136–0.741)	0.009

*Note:* Model 1: unadjusted. Model 2: adjusted gender, race, education level, marital status, body mass index, smoking, poverty-to-income ratio, physical activity, hypertension, diabetes, dyslipidemia, liver disease, cardiovascular disease, seen a mental health professional in the prior 12 months, antidepression drug, energy, protein, total fat, iron, calcium, potassium, sodium, saturated fatty acids, total monounsaturated fatty acids, total polyunsaturated fatty acids, vitamin B_6_, and caffeine.

Abbreviations: CI = confidence interval, OR = odds ratio.

**Table 4:** Subgroup analysis.

**Variables**	**Depression symptoms**	**OR (95% CI)**	**p**	**OR (95% CI)**	**p**
		Cancer = no		Cancer = yes	

Total fiber	None/mild	0.992 (0.982–1.002)	0.132	0.997 (0.968–1.027)	0.831
	None/moderate	0.975 (0.958–0.993)	0.006	0.994 (0.941–1.050)	0.830
	None/moderately severe	0.961 (0.934–0.989)	0.007	0.965 (0.897–1.039)	0.338
	None/severe	0.955 (0.918–0.994)	0.026	0.956 (0.859–1.064)	0.411

Cereal fiber	None/mild	1.007 (0.984–1.031)	0.540	1.033 (0.981–1.089)	0.212
	None/moderate	0.999 (0.940–1.061)	0.964	1.043 (0.973–1.119)	0.230
	None/moderately severe	0.851 (0.748–0.969)	0.015	0.720 (0.419–1.236)	0.230
	None/severe	0.895 (0.673–1.189)	0.439	0.955 (0.687–1.327)	0.781

Vegetable fiber	None/mild	1.011 (0.975–1.047)	0.556	0.901 (0.739–1.099)	0.300
	None/moderate	0.920 (0.848–0.998)	0.044	0.851 (0.683–1.060)	0.148
	None/moderately severe	0.928 (0.808–1.067)	0.292	0.110 (0.029–0.419)	0.001
	None/severe	0.977 (0.863–1.105)	0.706	0.404 (0.136–1.196)	0.101

Fruit fiber	None/mild	1.007 (0.961–1.055)	0.761	1.023 (0.866–1.209)	0.785
	None/moderate	0.939 (0.789–1.118)	0.477	0.891 (0.568–1.397)	0.610
	None/moderately severe	1.012 (0.875–1.169)	0.876	0.665 (0.236–1.871)	0.436
	None/severe	0.637 (0.413–0.983)	0.042	0.046 (0.001–2.080)	0.112

		CVD = no		CVD = yes	

Total fiber	None/mild	0.994 (0.984–1.004)	0.224	0.989 (0.961–1.019)	0.466
	None/moderate	0.976 (0.960–0.992)	0.004	0.987 (0.944–1.032)	0.565
	None/moderately severe	0.963 (0.935–0.993)	0.017	0.960 (0.916–1.006)	0.088
	None/severe	0.958 (0.917–0.909)	0.049	0.981 (0.900–1.069)	0.652

Cereal fiber	None/mild	1.007 (0.983–1.031)	0.557	1.049 (0.978–1.124)	0.177
	None/moderate	1.012 (0.958–1.069)	0.669	0.806 (0.600–1.081)	0.148
	None/moderately severe	0.821 (0.718–0.938)	0.004	0.954 (0.761–1.197)	0.682
	None/severe	0.906 (0.697–1.178)	0.458	0.871 (0.558–1.359)	0.539

Vegetable fiber	None/mild	1.008 (0.974–1.044)	0.639	0.914 (0.746–1.121)	0.385
	None/moderate	0.902 (0.834–0.975)	0.010	0.992 (0.792–1.242)	0.942
	None/moderately severe	0.891 (0.752–1.056)	0.182	1.012 (0.823–1.245)	0.908
	None/severe	0.955 (0.821–1.111)	0.548	0.893 (0.677–1.179)	0.421

Fruit fiber	None/mild	0.990 (0.939–1.044)	0.722	1.146 (0.963–1.363)	0.124
	None/moderate	0.933 (0.791–1.101)	0.409	0.839 (0.513–1.374)	0.482
	None/moderately severe	1.003 (0.859–1.172)	0.966	0.907 (0.608–1.353)	0.630
	None/severe	0.541 (0.273–1.071)	0.077	0.622 (0.294–1.314)	0.211

		Diabetes = no		Diabetes = yes	

Total fiber	None/mild	0.996 (0.973–1.019)	0.724	0.994 (0.985–1.003)	0.207
	None/moderate	0.944 (0.910–0.978)	0.002	0.986 (0.969–1.002)	0.083
	None/moderately severe	0.973 (0.910–1.040)	0.411	0.962 (0.936–0.989)	0.007
	None/severe	0.935 (0.865–1.010)	0.087	0.960 (0.916–1.005)	0.081

Cereal fiber	None/mild	0.996 (0.938–1.057)	0.895	1.011 (0.988–1.034)	0.355
	None/moderate	0.993 (0.902–1.094)	0.884	0.987 (0.919–1.060)	0.723
	None/moderately severe	0.794 (0.600–1.051)	0.106	0.839 (0.735–0.957)	0.009
	None/severe	0.864 (0.615–1.213)	0.394	0.913 (0.688–1.211)	0.523

Vegetable fiber	None/mild	0.965 (0.852–1.093)	0.571	1.006 (0.969–1.044)	0.755
	None/moderate	0.806 (0.599–1.083)	0.151	0.916 (0.845–0.992)	0.032
	None/moderately severe	0.870 (0.629–1.203)	0.395	0.920 (0.787–1.076)	0.292
	None/severe	0.728 (0.417–1.270)	0.260	0.979 (0.869–1.102)	0.719

Fruit fiber	None/mild	0.960 (0.774–1.192)	0.710	1.015 (0.969–1.063)	0.517
	None/moderate	0.572 (0.390–0.839)	0.005	0.986 (0.851–1.143)	0.851
	None/moderately severe	0.784 (0.596–1.031)	0.081	1.027 (0.880–1.199)	0.729
	None/severe	0.741 (0.326–1.685)	0.470	0.500 (0.262–0.955)	0.036

		Dyslipidemia = no		Dyslipidemia = yes	

Total fiber	None/mild	0.996 (0.985–1.007)	0.482	0.985 (0.969–1.002)	0.078
	None/moderate	0.977 (0.956–0.999)	0.041	0.973 (0.948–0.909)	0.048
	None/moderately severe	0.966 (0.937–0.996)	0.029	0.962 (0.920–1.005)	0.084
	None/severe	0.974 (0.935–1.014)	0.198	0.937 (0.868–1.012)	0.097

Cereal fiber	None/mild	1.000 (0.962–1.040)	0.986	1.014 (0.979–1.051)	0.421
	None/moderate	1.026 (0.952–1.105)	0.497	1.001 (0.945–1.061)	0.974
	None/moderately severe	0.844 (0.735–0.968)	0.016	0.854 (0.695–1.050)	0.133
	None/severe	0.485 (0.189–1.242)	0.130	0.989 (0.861–1.137)	0.878

Vegetable fiber	None/mild	1.019 (0.965–1.076)	0.495	0.989 (0.923–1.059)	0.741
	None/moderate	0.880 (0.780–0.994)	0.040	0.909 (0.806–1.024)	0.116
	None/moderately severe	0.853 (0.689–1.055)	0.141	0.998 (0.830–1.201)	0.987
	None/severe	1.065 (0.912–1.244)	0.424	0.890 (0.756–1.048)	0.159

Fruit fiber	None/mild	1.054 (0.999–1.112)	0.056	0.947 (0.842–1.066)	0.364
	None/moderate	0.979 (0.782–1.226)	0.850	0.863 (0.700–1.064)	0.165
	None/moderately severe	1.068 (0.909–1.256)	0.420	0.825 (0.673–1.011)	0.064
	None/severe	0.473 (0.136–1.650)	0.237	0.344 (0.131–0.899)	0.030

		Hypertension = no		Hypertension = yes	

Total fiber	None/mild	0.993 (0.982––1.005)	0.239	0.994 (0.976–1.012)	0.483
	None/moderate	0.982 (0.962–1.003)	0.088	0.966 (0.941–0.992)	0.011
	None/moderately severe	0.976 (0.946–1.008)	0.139	0.948 (0.909–0.989)	0.013
	None/severe	0.966 (0.927–1.007)	0.098	0.949 (0.876–1.028)	0.195

Cereal fiber	None/mild	1.008 (0.982–1.034)	0.564	1.017 (0.972–1.064)	0.460
	None/moderate	1.033 (0.980–1.089)	0.228	0.894 (0.814–0.982)	0.019
	None/moderately severe	0.827 (0.706–0.968)	0.018	0.852 (0.724–1.003)	0.054
	None/severe	0.546 (0.312–0.956)	0.034	1.003 (0.841–1.196)	0.977

Vegetable fiber	None/mild	1.020 (0.980–1.062)	0.323	0.969 (0.898–1.045)	0.411
	None/moderate	0.944 (0.865–1.030)	0.194	0.837 (0.726–0.964)	0.014
	None/moderately severe	0.749 (0.553–1.013)	0.061	0.989 (0.865–1.132)	0.876
	None/severe	0.966 (0.821–1.136)	0.673	0.907 (0.704–1.168)	0.446

Fruit fiber	None/mild	0.999 (0.941–1.060)	0.973	1.021 (0.955–1.092)	0.533
	None/moderate	1.003 (0.857–1.173)	0.973	0.776 (0.608–0.990)	0.041
	None/moderately severe	1.034 (0.873–1.226)	0.694	0.903 (0.717–1.138)	0.384
	None/severe	0.685 (0.346–1.356)	0.274	0.474 (0.250–0.898)	0.022

*Note:* Adjusted gender, race, education level, marital status, body mass index, smoking, poverty-to-income ratio, physical activity, hypertension (was not adjusted in hypertension subgroup analysis), diabetes (was not adjusted in diabetes subgroup analysis), dyslipidemia (was not adjusted in dyslipidemia subgroup analysis), liver disease, CVD (was not adjusted in CVD subgroup analysis), seen a mental health professional in the prior 12 months, antidepression drug, energy, protein, total fat, iron, calcium, potassium, sodium, saturated fatty acids, total monounsaturated fatty acids, total polyunsaturated fatty acids, vitamin B_6_, and caffeine.

Abbreviations: CI = confidence interval, CVD = cardiovascular disease, OR = odds ratio.

## Data Availability

The datasets generated and/or analyzed during the current study are available in the NHANES database (https://wwwn.cdc.gov/nchs/nhanes/).

## References

[1] Ljungberg T., Bondza E., Lethin C. (2020). Evidence of the importance of dietary habits regarding depressive symptoms and depression. *International Journal of Environmental Research and Public Health*.

[2] COVID-19 Mental Disorders Collaborators (2021). Global prevalence and burden of depressive and anxiety disorders in 204 countries and territories in 2020 due to the COVID-19 pandemic. *The Lancet*.

[3] Dean J., Keshavan M. (2017). The neurobiology of depression: an integrated view. *Asian Journal of Psychiatry*.

[4] Torabynasab K., Shahinfar H., Payandeh N., Jazayeri S. (2023). Association between dietary caffeine, coffee, and tea consumption and depressive symptoms in adults: a systematic review and dose-response meta-analysis of observational studies. *Frontiers in Nutrition*.

[5] Huang R., Wang K., Hu J. (2016). Effect of probiotics on depression: a systematic review and meta-analysis of randomized controlled trials. *Nutrients*.

[6] Kris-Etherton P. M., Petersen K. S., Hibbeln J. R. (2021). Nutrition and behavioral health disorders: depression and anxiety. *Nutrition Reviews*.

[7] Li Y., Lv M. R., Wei Y. J. (2017). Dietary patterns and depression risk: a meta-analysis. *Psychiatry Research*.

[8] Xu Y., Zeng L., Zou K. (2021). Role of dietary factors in the prevention and treatment for depression: an umbrella review of meta-analyses of prospective studies. *Translational Psychiatry*.

[9] Albenberg L. G., Wu G. D. (2014). Diet and the intestinal microbiome: associations, functions, and implications for health and disease. *Gastroenterology*.

[10] Dinan T. G., Cryan J. F. (2017). The microbiome-gut-brain axis in health and disease. *Gastroenterology Clinics of North America*.

[11] Liu Y., Ju Y., Cui L. (2021). Association between dietary fiber intake and incidence of depression and anxiety in patients with essential hypertension. *Nutrients*.

[12] Xia Y., Liu Y., Zhang S. (2021). Associations between different types and sources of dietary fibre intake and depressive symptoms in a general population of adults: a cross-sectional study. *The British Journal of Nutrition*.

[13] Xu H., Li S., Song X., Li Z., Zhang D. (2018). Exploration of the association between dietary fiber intake and depressive symptoms in adults. *Nutrition*.

[14] Miki T., Eguchi M., Kurotani K. (2016). Dietary fiber intake and depressive symptoms in Japanese employees: the Furukawa nutrition and health study. *Nutrition*.

[15] Fan Z., Gong X., Xu H. (2022). Gender differences in the associations between tobacco smoke exposure and depressive symptoms among U.S. adults: NHANES 2007-2018. *Journal of Psychiatric Research*.

[16] Kroenke K., Spitzer R. L., Williams J. B. W. (2001). The PHQ-9: validity of a brief depression severity measure. *Journal of General Internal Medicine*.

[17] Parikh N. S., Salehi Omran S., Kamel H., Elkind M. S. V., Willey J. (2020). Symptoms of depression and active smoking among survivors of stroke and myocardial infarction: an NHANES analysis. *Preventive Medicine*.

[18] Voelker J., Wang K., Tang W. (2021). Association of depression symptom severity with short-term risk of an initial hospital encounter in adults with major depressive disorder. *BMC Psychiatry*.

[19] Zhao H., Yang A., Mao L., Quan Y., Cui J., Sun Y. (2020). Association between dietary fiber intake and non-alcoholic fatty liver disease in adults. *Frontiers in Nutrition*.

[20] Schmidhuber J., Sur P., Fay K. (2018). The global nutrient database: availability of macronutrients and micronutrients in 195 countries from 1980 to 2013. *The Lancet Planetary Health*.

[21] Chrzastek Z., Guligowska A., Piglowska M., Soltysik B., Kostka T. (2022). Association between sucrose and fiber intake and symptoms of depression in older people. *Nutritional Neuroscience*.

[22] Khayyatzadeh S. S., Omranzadeh A., Miri-Moghaddam M. M. (2021). Dietary antioxidants and fibre intake and depressive symptoms in Iranian adolescent girls. *Public Health Nutrition*.

[23] Fatahi S., Matin S. S., Sohouli M. H. (2021). Association of dietary fiber and depression symptom: a systematic review and meta-analysis of observational studies. *Complementary Therapies in Medicine*.

[24] Saghafian F., Sharif N., Saneei P. (2021). Consumption of dietary fiber in relation to psychological disorders in adults. *Frontiers in Psychiatry*.

[25] Sun Y., Cheng L., Zeng X. (2021). The intervention of unique plant polysaccharides - dietary fiber on depression from the gut-brain axis. *International Journal of Biological Macromolecules*.

[26] Swann O. G., Kilpatrick M., Breslin M., Oddy W. H. (2020). Dietary fiber and its associations with depression and inflammation. *Nutrition Reviews*.

[27] Levis B., Benedetti A., Thombs B. D., DEPRESsion Screening Data (DEPRESSD) Collaboration (2019). Accuracy of Patient Health Questionnaire-9 (PHQ-9) for screening to detect major depression: individual participant data meta-analysis. *BMJ*.

[28] Li W., Ruan W., Peng Y., Lu Z., Wang D. (2021). Associations of socioeconomic status and sleep disorder with depression among US adults. *Journal of Affective Disorders*.

