# Supplementary material for: Association Between Dietary Fiber and the Severity of Depression Symptoms
Source: Behav Neurol. 2024 Oct 28;2024:5510304. doi: 10.1155/2024/5510304 (PMC11535533; doi:10.1155/2024/5510304)
Supplement: Supporting Information — Additional supporting information can be found online in the Supporting Information section. Table S1. Identify potential confounding factors. [file 5510304.f1.docx]

**Supplemental Table 1 Identify potential confounding factors**

| Variables | Beta (95%CI) | *P* |
| --- | --- | --- |
| Age | -0.001 (-0.001, 0.01) | 0.128 |
| Gender |  |  |
| Male | Ref |  |
| Female | 0.143 (0.121, 0.165) | <0.001 |
| Race |  |  |
| White | Ref |  |
| Black | 0.065 (0.033, 0.096) | <0.001 |
| Others | 0.032 (0.004, 0.061) | 0.027 |
| Education level |  |  |
| Less than high school | Ref |  |
| Above high school | -0.197 (-0.239, -0.156) | <0.001 |
| Unknown | -0.183 (-0.249, -0.117) | <0.001 |
| Marital status |  |  |
| Married | Ref |  |
| Unmarried | 0.194 (0.172, 0.216) | <0.001 |
| Unknown | 0.072 (0.021, 0.124) | 0.007 |
| PIR |  |  |
| <1 | Ref |  |
| ≥1 | -0.311 (-0.348, -0.274) | <0.001 |
| Unknown | -0.277 (-0.322, -0.232) | <0.001 |
| Smoking |  |  |
| No | Ref |  |
| Yes | 0.176 (0.152, 0.200) | <0.001 |
| Drinking | |  |
| No | Ref |  |
| Yes | 0.018 (-0.005, 0.041) | 0.134 |
| Unknown | 0.026 (-0.068, 0.120) | 0.592 |
| BMI | 0.010 (0.008, 0.012) | <0.001 |
| Physical activity |  |  |
| <750 | Ref |  |
| ≥750 | -0.132 (-0.157, -0.106) | <0.001 |
| Unknown | 0.049 (-0.362, 0.461) | 0.815 |
| Diabetes |  |  |
| No | Ref |  |
| Yes | 0.182 (0.141, 0.223) | <0.001 |
| Hypertension | |  |
| No | Ref |  |
| Yes | 0.146 (0.118, 0.175) | <0.001 |
| Dyslipidemia | |  |
| No | Ref |  |
| Yes | 0.080 (0.057, 0.104) | <0.001 |
| Unknown | 0.051 (0.017, 0.084) | 0.004 |
| Cancer or malignancy |  |  |
| No | Ref |  |
| Yes | 0.016 (-0.023, 0.055) | 0.420 |
| Unknown | -0.010 (-0.060, 0.040) | 0.697 |
| Liver condition | |  |
| No | Ref |  |
| Yes | 0.315 (0.235, 0.395) | <0.001 |
| Unknown | 0.018 (-0.037, 0.073) | 0.517 |
| CVD |  |  |
| No | Ref |  |
| Yes | 0.268 (0.217, 0.318) | <0.001 |
| Seen mental health professional in the prior 12 months |  |  |
| No | Ref |  |
| Yes | 0.641 (0.573, 0.709) | <0.001 |
| Energy | -0.000 (-0.000, -0.000) | <0.001 |
| Protein | -0.002 (-0.002, -0.001) | <0.001 |
| Carbohydrate | -0.000 (-0.000, -0.000) | 0.028 |
| Total fat | -0.001 (-0.001, -0.01) | <0.001 |
| Iron | -0.005 (-0.007, -0.004) | <0.001 |
| Calcium | -0.000 (-0.000, -0.000) | <0.001 |
| Potassium | -0.000 (-0.000, -0.000) | <0.001 |
| Sodium | -0.000 (-0.000, -0.000) | <0.001 |
| Total saturated fatty acids | -0.001 (-0.002, -0.01) | 0.043 |
| MUFA | -0.002 (-0.003, -0.001) | <0.001 |
| PUFA | -0.003 (-0.004, -0.002) | <0.001 |
| Vitamin B_6_ | -0.016 (-0.027, -0.006) | 0.003 |
| Vitamin B_12_ | -0.000 (-0.002, 0.002) | 0.811 |
| Caffeine | 0.000 (0.000, 0.000) | <0.001 |

Note: BMI=body mass index; PIR=poverty-to-income ratio; CVD=cardiovascular disease; PUFA=total polyunsaturated fatty acids; MUFA=total monounsaturated fatty acids.
